# A Review of *Coccidioides* Research, Outstanding Questions in the Field, and Contributions by Women Scientists

**DOI:** 10.1007/s40588-021-00173-9

**Published:** 2021-08-02

**Authors:** Morgan E. Gorris, Marley C. Caballero Van Dyke, Adrienne Carey, Paris S. Hamm, Heather L. Mead, Jessie K. Uehling

**Affiliations:** 1 Los Alamos National Laboratory, Information Systems and Modeling & Center for Nonlinear Studies, Los Alamos, NM USA; 2 Pathogen and Microbiome Institute, Northern Arizona University, Flagstaff, AZ USA; 3 Division of Infectious Diseases, University of Utah School of Medicine, Salt Lake City, UT USA; 4 Department of Biology, University of New Mexico, Albuquerque, NM USA; 5 Department of Botany and Plant Pathology, Oregon State University, Corvallis, OR USA

**Keywords:** *Coccidioides*, Mycology, Environment, Genetics, Host response, Valley fever

## Abstract

**Purpose of Review:**

Coccidioidomycosis is an infectious disease that gained clinical significance in the early 20th century. Many of the foundational contributions to coccidioidomycosis research, including the discovery of the fungal disease agent, Coccidioides spp., were made by women. We review recent progress in Coccidioides research and big questions remaining in the field, while highlighting some of the contributions from women.

**Recent Findings:**

New molecular-based techniques provide a promising method for detecting Coccidioides, which can help determine the dominate reservoir host and ideal environmental conditions for growth. Genetic and genomic analyses have allowed an understanding of population structure, species level diversity, and evolutionary histories. We present a current, comprehensive genome list, where women contributed many of these entries. Several efforts to develop a coccidioidomycosis vaccine are underway.

**Summary:**

Women continue to pioneer research on Coccidioides, including the relationships between the fungi and the environment, genetics, and clinical observations. Significant questions remain in the field of Coccidioides, including the main host reservoir, the relationships between genotypic and phenotypic variation, and the underlying cause for chronic clinical coccidioidomycosis cases.

## Introduction

Since the early twentieth century when coccidioidomycosis gained clinical significance, women have made significant contributions to the advancements in understanding this disease. Many of the foundational studies on the environmental, genetic, and clinical aspects of coccidioidomycosis have been led by women, including the identification of the causative fungal pathogen, *Coccidioides* spp. In 1934, Dr. Myrnie Gifford, an assistant health officer for Kern County, California, was the first physician to investigate a Californian disease called “San Joaquin Valley fever” (the origins of its colloquial name, Valley fever). She proved that *Coccidioides* was the causative agent for Valley fever and continued to observe clinical and epidemiological outcomes throughout her career [1–4]. This initial connection between the causative agent and resultant *Coccidioides* infection provided a clear target for future research endeavors and immediately helped raise awareness about the risk for coccidioidomycosis, especially in California’s Central Valley.

As the foundational knowledge about *Coccidioides* continued to grow, women approached the need to understand what communities may be at risk for coccidioidomycosis infection. Starting in the mid-1940s, Dr. Phyllis Edwards, in collaboration with Dr. Caroll Palmer, conducted a seminal seroprevalence study among young, healthy volunteers to establish the first *Coccidioides* endemic map [5]. Conducted between 1945 and 1951, over 110,000 Navy recruits, student nurses, and college students were enrolled in this coccidioidin skin testing study that led to the first known map of disease distribution across the USA. Prior to this research, little was known about the disease outside of specific areas of California, Arizona, New Mexico, and Texas [6–8].

In addition to determining where coccidioidomycosis infection is likely to occur, women have also significantly contributed to understanding the clinical outcomes of this disease. While the majority of coccidioidomycosis cases are asymptomatic, the predominant manifestation is primary pulmonary disease in the form of pneumonia [9]. Overall, the disease tends to impact men more than women [10]. However, in recent years, Dr. Rebecca Sunenshine and colleagues analyzed case data in Arizona from 2009 to 2015 and found a slight female predominance [11]. Race is also a factor in increased risk of infection; BIPOC (black, Indigenous, and people of color) women are at greater risk than white women to develop disseminated disease (organ involvement outside of the lungs) and require hospitalization [12, 13]. Accounting for level of exposure, race alone does not confer an inherent increased risk to inhale the arthroconidia of *Coccidioides*; occupation, access to and quality of health care, and a variety of research-related bias contribute to this paradigm [12]. Pregnancy is not only a risk factor for coccidioidomycosis, but also for more severe manifestations [14–17]. Acquisition of the disease during pregnancy, especially during the third trimester and postpartum period, increases the likelihood of poor outcomes, including death [14, 18, 19]. Depending on the timing of acquisition during pregnancy, the percent likelihood of disease dissemination ranges from 10 to 96% [15, 17].

**Figure 1.**
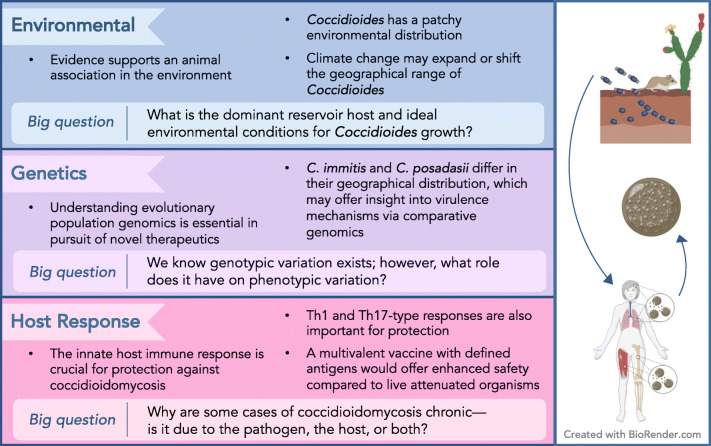
Overview of the paper. Here we highlight the takeaway message from each section along with unanswered questions in the field of *Coccidioides* research.

Though sometimes overlooked or under cited for their contributions, women have made and continue to make significant achievements in understanding *Coccidioides* and coccidioidomycosis. We provide an overview of some of the contributions of women to the field of *Coccidioides*, emphasizing past contributions, current research, and big questions remaining for the field (Figure 1). In addition to specifically naming the women associated with certain contributions, we endorse the claims made throughout our review using many studies led and supported by women.

### Environment

Several questions remain regarding the environmental conditions conducive to the presence of *Coccidioides* and areas of enhanced risk for contracting coccidioidomycosis, including the dominant reservoir host, favorable soil properties, and ideal climate conditions [20]. Answering these questions has been difficult due to the challenge of acquiring environmental samples of *Coccidioides*. New molecular-based techniques provide a promising method for detection, which could accelerate research in this area.

### The *Coccidioides* Host Reservoir

An exact host reservoir for *Coccidioides* has partially eluded researchers. It has become evident over recent decades that *Coccidioides* depends on animal species in order to maintain its life cycle [21, 22••]. Comparative genomic studies emphasized a shift from plant tissue-associated genes to animal tissue-associated genes by discovering a significant decrease of genes involved in cell wall degradation (cellulase, tannase, cutinase, and pectin lyase) paired with an expansion of proteases and keratinases suggesting a nutritional association with mammals [23, 24]. A revival of research from the 1940s [25, 26] has steered researchers’ attention to rodents and their burrows as the main hypothesis for a dominant host reservoir. Multiple studies have exhibited a higher percentage recovery of *Coccidioides* from soil collected from rodent burrows than from surrounding topsoil [27–29]. Early on, an experiment performed by Maddy and Crecelius (1967) buried mice that were experimentally infected with *Coccidioides* in endemic soils that had previously failed to produce cultures of *C. immitis* for 3 years. Five months after the burial, that soil produced a positive culture and remained positive for 6 subsequent years of investigation, demonstrating sporulation of *Coccidioides* in the carcasses of dead rodents [30]. Even if rodents are the correct hypothesized dominant reservoir, Rodentia is still the most diverse order within Mammalia, encompassing 2,590 species within 521 genera [31]. Very little research has been conducted to pinpoint a dominant host genus or species. It is possible, if not likely, that *C. immitis* and *C. posadasii* may be specialized for different host reservoirs, which could vary by geographical region. One species of interest for further investigation in the southwestern US is the desert woodrat (*Neotoma lepida*), whose presence significantly overlaps with human coccidioidomycosis cases [32].

Although current hypotheses suggest that rodents are the predominant host reservoir, *Coccidioides* has been detected in a diverse array of wild mammals including bats [33], armadillos [34], sea lions and sea otters [35], llamas and alpacas [36], and numerous non-native zoo animals [21]. The presence of *Coccidioides* in both terrestrial and marine mammals, as well as volant mammals, suggests that fungal dispersion could take multiple avenues with the migration of their commensal hosts. *Coccidioides*, present even in low abundance in the lungs [37], may be adapted to proliferate on hair, skin, nails/hooves, and bones of carcasses [38]. This supports an eventual shift in the fungal burden from the host back to the soil; in turn, this maintains the modality of *Coccidioides* being dispersed by wind or soil disturbance and thus the inhalation of arthroconidia into future hosts. This cycle, in part, could supplement the explanation of a geographic range expansion of *Coccidioides* associated with human migration [39].

### Soil, Environmental, and Climatic Conditions

In addition to the presence of important host reservoirs, certain soil conditions may be favorable habitats for *Coccidioides*. Drs. Ann Elconin and Margret Egeberg highlighted the significance of studying physical soil properties such as alkalinity and salinity, which may explain some of the heterogeneity in the presence of *Coccidioides* within each microenvironment [28, 40]. However, recent correlations between soil properties and the presence of *Coccidioides* have been inconsistent across studies, making it challenging to draw definitive conclusions. For example, soil properties have been the same where both positive and negative *Coccidioides* samples were collected [41]. Dr. Antje Lauer and colleagues continue to explore the potential for alkaline soils and the associated vegetation, as well as sandy loam soil textures, to be a favorable soil microenvironment for *Coccidioides* [42, 43]. In addition to abiotic factors, biotic factors such as microbial competition may also be an important determinant for the presence of *Coccidioides* [21]. Parsing the differences in preferential abiotic and biotic soil environments between *C. immitis* and *C. posadasii* would help disentangle any differences in the geographical distribution of these pathogens.

Climate conditions also likely determine the timing and amount of *Coccidioides* growth within endemic soils. *Coccidioides* is thought to proliferate following wet conditions; then if stressed by hot and dry conditions, it will autolyze into arthroconidia that may be easily dispersed by the wind [44]. Although it has not been possible to study this mechanism at the microbial level, human cases of coccidioidomycosis have been correlated with lagged climatic conditions, where cases were higher following dry and warm months [45]. In southern Arizona, temperatures in the preceding season have had a significant positive relationship with coccidioidomycosis incidence [45]. In the San Joaquin Valley of California, autumn levels of human coccidioidomycosis incidence have been higher following cooler and wetter winter and spring months [46, 47]. Not only do climate conditions affect the seasonal pattern of disease incidence, but they can also modulate the interannual variation in the number of disease cases [45, 46], though this relationship is less clear. Taken in combination, considering the effects of climate conditions with demographic and health risk factors provides a method of calculating a vulnerability index for coccidioidomycosis [48], which can help identify communities most at risk for contracting this disease.

### The Geographical Distribution of *Coccidioides*

Together, the host reservoirs, soil environment, and climate conditions likely structure the environmental niche and therefore geographical distribution of *Coccidioides*. Dr. Meritxell Riquelme’s lab has been at the forefront of examining where *Coccidioides* lives in the environment. This is imperative to understanding the risk of coccidioidomycosis, especially if the geographical distribution shifts in response to climate change [49]. Soil samples positive for *Coccidioides*, human coccidioidomycosis case data, and important environmental drivers paired with ecological niche models have made it possible to create high-resolution estimates of the *Coccidioides* endemic region [32, 50, 51••]. Using human coccidioidomycosis data as a proxy for *Coccidioides* presence, Dr. Morgan Gorris and colleagues predict that by the end of the twenty-first century, warming temperatures across the dry, western US may cause the endemic region to expand north [51••]. This may cause a large increase in the number of people at risk for contracting coccidioidomycosis, the subsequent number of disease cases, and the financial burden of this disease [52]. In contrast, a separate analysis using *Coccidioides* presence data in a niche model found that by 2070, the geographical distribution of *Coccidioides* may contract, but the habitat suitability within already suitable locations will increase [32]. Continued disease surveillance and soil sampling will help to further resolve the ecological niche of *Coccidioides* and discrepancies between estimates, especially as we identify new endemic areas, such as Washington State [53, 54]. By identifying differences between *Coccidioides* species, like thermotolerance [55•], we can also determine if there is a niche unique to each species. This will help to delineate the geographical distribution of each species, which may be different than originally thought; for example, *C. immitis*, originally deemed the “California species,” has also been found in New Mexico, Utah, and Washington [53, 56, 57]. Understanding the distribution of *Coccidioides* will help with assessing human risk for coccidioidomycosis and identifying locations prone to disease outbreaks from activities with large soil disturbance like agriculture, construction, or natural hazards like wildfires, dust storms, and landslides from earthquakes [41, 58–62].

In many ways, future research of *Coccidioides* is dependent on acquiring environmental samples to supplement clinical and veterinary samples. Although *C. immitis* can be cultured directly from soils, it has proven difficult with one study only obtaining 0.55% (4 out of 720) success in a known endemic range of California [63]. For *C. posadasii*, which has not been directly cultured from soil, Dr. Bridget Barker and collaborators demonstrated that detection is possible using BALB/c mice as biosensors in soils near Tucson, Arizona, with a 8.9% positive detection rate [64]. Intraperitoneal inoculation into mice was also successful in isolating *C. posadasii* from six out of 24 (25%) soil samples from Brazil [65]. A promising, new detection tool developed by Drs. Jolene Bowers and Bridget Barker is the CocciENV real-time PCR assay, which provides a robust way to test a large number of soils for *Coccidioides* DNA [66]. Although not commonly used in practice, molecular-based technologies exist to differentiate the two species, which may become more essential as we tease apart phenotypic differences [56, 67].

### *Coccidioides* Genetics

Since the early 1990s, scientists have used classic genetics, and later genomics, to study *Coccidioides*. Notable contributing researchers include Dr. Bridget Barker, Dr. Chiung-Yu Hung, Dr. Clarisa Nobile, and Dr. Emily Whiston. Together, they and their colleagues have produced a seminal body of research literature identifying key virulence genes and elucidating the role of genetic systems. In parallel, they have utilized and developed a genetic tool kit including genome sequences and editing tools to continue exploring the molecular underpinnings of *Coccidioides* virulence.

Phenotypic and molecular studies have long suggested that *Coccidioides* populations differ by region and disease manifestation. There are at least two species of *Coccidioides* in Western North America, Central America, and South America and the genomic variation within isolates within those sub-populations that can be leveraged to answer long-standing questions about the variable effects of coccidioidomycosis isolates in humans and animals.

### Populations and Variance in Genomes

Genetic and genomic analyses have allowed an understanding of population structure, species level diversity, and evolutionary histories. Better understanding the molecular diversity of *Coccidioides* and how past evolutionary selective pressures have resulted in current molecular virulence mechanisms will greatly expedite novel therapeutic strategies for treating *Coccidioides*. Though *C. posadasii* was originally described in 1896, it was not until 2002 when microsatellite and ITS sequence data were available that *C. immitis* and *C. posadasii* were differentiated [39]. Since then, we have gained appreciation for substantial intraspecific *C. posadasii* population structure in Texas, Mexico, and South America [22••], and that Arizona has the most diverse isolates of *C. posadasii.* This is reflected in the asymmetric-genome sequence availability of *C. posadasii* and *C. immitis* isolates, many of which were contributed by women (Table 1). Whole-genome sequencing of over 30 new isolates [68] shows that *Coccidioides* likely originated in the Sonoran Desert.

**Table 1.** Available genome resources for *Coccidioides*

Species	SRA/NCBI study^a^	Sample name^b^	Publication^c^
*C. immitis*	SRP074212	B0727_Argentina	[68]
*C. immitis*	SRP148748	B11080	[69]
*C. immitis*	SRP148748	B11518	[70]
*C. immitis*	SRP148748	B11587	[70]
*C. immitis*	SRP148748	B11863	[70]
*C. immitis*	SRP148748	B11873	[70]
*C. immitis*	SRP148748	B12398	[70]
*C. immitis*	SRP148748	B12495	[70]
*C. immitis*	SRP148748	B12496	[70]
*C. immitis*	SRP148748	B13956	[70]
*C. immitis*	SRP074212	Coahuila_1	[68, 71]
*C. immitis*	SRP074212	Guerrero_1, RMSCC3479	[68, 71]
*C. immitis*	PRJNA17355	H538.4	[72]
*C. immitis*	SRP074212	Michoacan_2, RMSCC3476	[68, 71]
*C. immitis*	PRJNA17713	RMSCC2394	[69]
*C. immitis*	PRJNA17761	RMSCC3703	[69]
*C. immitis*	GCA_000149335.2, AAEC00000000.3, PRJNA16822 PRJNA169242	RS	[55•, 69, 73]
*C. immitis*	SRP042092	San_Diego_1, RMSCC3706	[53, 55•]
*C. immitis*	SRP042092	San_Joaquin_Valley_11, RMSCC2281	[53, 55•, 70]
*C. immitis*	SRP042092	San_Joaquin_Valley_2, RMSCC22012	[53, 55•]
*C. immitis*	SRP042092	San_Joaquin_Valley_5, RMSCC2268	[53, 55•, 70]
*C. immitis*	SRP042092	San_Joaquin_Valley_6, RMSCC2269	[53, 55•, 70]
*C. immitis*	SRP042092	San_Joaquin_Valley_9, RMSCC2279	[53, 55•, 70]
*C. immitis*	SRP074212	SJV_1, RMSCC2009	[68, 70]
*C. immitis*	SRP074212	SJV_10, RMSCC2280	[55•, 68, 70]
*C. immitis*	SRP074212	SJV_3, RMSCC22015	[55•, 68, 70]
*C. immitis*	SRP074212	SJV_4, RMSCC22017	[55•, 68, 70]
*C. immitis*	SRP074212	SJV_7, RMSCC2273	[55•, 68, 70]
*C. immitis*	SRP074212	SJV_8, RMSCC2277	[55•, 68, 70]
*C. immitis*	SRP042092	WA_202, CDC_202, B10992	[53, 70, 71]
*C. immitis*	SRP042092	WA_205, CDC_205, B10988	[53, 70, 71]
*C. immitis*	SRP042092	WA_211, CDC_211, B10992	[53, 69–71, 128]
*C. immitis*	SRP042092	WA_212, BB10996	[53, 70, 71]
*C. immitis*	SRP042092	Washington_1, B10637	[53, 70]
*C. posadasii*	SRP135537	2566, Cp_6	[71]
*C. posadasii*	SRP135537	34698, Cp_4	[71]
*C. posadasii*	SRP135537	3490, Cp_8	[71]
*C. posadasii*	SRP135537	3796, Cp_5	[71]
*C. posadasii*	SRP135537	4542, Cp_3	[71]
*C. posadasii*	SRP135537	4545-MICE, Cp_2	[71]
*C. posadasii*	SRP135537	4545, Cp_1	[71]
*C. posadasii*	SRP074212	730332_Guatemala	[68, 71]
*C. posadasii*	SRP074212	730333_Guatelama	[68, 71]
*C. posadasii*	SRP074212	730334_Guatemala	[68, 71]
*C. posadasii*	SRP074212	B0858_Guatemala	[68, 71]
*C. posadasii*	SRP074212	B10757_Nevada	[68, 71]
*C. posadasii*	SRP074212	B10813_Texas	[68, 70, 71]
*C. posadasii*	SRP074212	B1249_Guatemala	[68, 71]
*C. posadasii*	SRP074212	B5773_Brazil	[68, 71]
*C. posadasii*	SRP135537	Beeville	[71]
*C. posadasii*	PRJNA472461	C735	[69, 23]
*C. posadasii*	PRJNA9616	C735 SOWgp	[23, 69]
*C. posadasii*	SRP074212	Coahuila_2, RMSCC3506	[55•, 68, 71]
*C. posadasii*	SRP074212	Colorado_Springs_1, VFC047	[68, 71]
*C. posadasii*	PRJNA17793	CPA0001	[69]
*C. posadasii*	PRJNA17795	CPA0020	[69]
*C. posadasii*	PRJNA17797	CPA0066	[69]
*C. posadasii*	PRJNA722304	Flagstaff_1, HS-I-000233	[55•]
*C. posadasii*	PRJNA722304	Flagstaff_2, HS-I-000234	[55•]
*C. posadasii*	PRJNA722304	Flagstaff_3, HS-I-000235	[55•]
*C. posadasii*	PRJNA722304	Flagstaff_4, HS-I-000449	[55•]
*C. posadasii*	PRJNA722304	Flagstaff_5, HS-I-000588	[55•]
*C. posadasii*	PRJNA722304	Flagstaff_6, HS-I-000718	[55•]
*C. posadasii*	PRJNA722304	Flagstaff_7, HS-I-000778	[55•]
*C. posadasii*	SRP074212	GT002_Texas	[68]
*C. posadasii*	SRP074212	GT017_Paraguay	[68]
*C. posadasii*	SRP135537	GT120, Cp_9	[71]
*C. posadasii*	SRP135537	GT162, Cp_10	[71]
*C. posadasii*	SRP135537	JTORRES, Cp_7	[71]
*C. posadasii*	SRP074212	Michoacan_1,RMSCC3472	[68, 71]
*C. posadasii*	SRP074212	Nuevo_Leon_1, RMSCC2343	[55•, 68, 71]
*C. posadasii*	SRP074212	Nuevo_Leon_2, RMSCC2346	[55•, 68, 71]
*C. posadasii*	SRP074212	Phoenix_1, ID02-184	[68, 71]
*C. posadasii*	SRP074212	Phoenix_2, ID03-517	[68, 71]
*C. posadasii*	SRP074212	Phoenix_3, ID03-584	[68, 71]
*C. posadasii*	SRP074212	Phoenix_4, ID03-587	[68, 71]
*C. posadasii*	SRP074212	Phoenix_5, 0204-3538	[68, 71]
*C. posadasii*	SRP074212	Phoenix_6, 0204-5786	[68, 71]
*C. posadasii*	SRP074212	Phoenix_7, 0204-7892	[68, 71]
*C. posadasii*	SRP074212	Phoenix_8, 0204-9888	[68, 71]
*C. posadasii*	SRP074212	Phoenix_9, 0205-5127	[68, 71]
*C. posadasii*	PRJNA17791	RMSCC1037	[69]
*C. posadasii*	PRJNA17785	RMSCC1038	[69]
*C. posadasii*	PRJNA17763	RMSCC2133	[69]
*C. posadasii*	PRJNA17783	RMSCC3488	[69]
*C. posadasii*	PRJNA17781	RMSCC3700	[69]
*C. posadasii*	SRP074212	San_Antonio_1	[68, 71]
*C. posadasii*	GCA_000170175.2	Silveira	[55•, 72, 129]
*C. posadasii*	SRP074212	Sonora_1, RMSCC3480	[55•, 68]
*C. posadasii*	SRP074212	Sonora_2, RMSCC3487	[55•, 68]
*C. posadasii*	SRP074212	Tucson_1, RMSCC3214	[68, 71]
*C. posadasii*	SRP074212	Tucson_10, RMSCC3252	[68, 71]
*C. posadasii*	SRP074212	Tucson_11, RMSCC3253	[68, 71]
*C. posadasii*	SRP074212	Tucson_12, RMSCC3262	[68, 71]
*C. posadasii*	SRP074212	Tucson_13, RMSCC3263	[68, 71]
*C. posadasii*	SRP074212	Tucson_14, RMSCC3268	[68, 71]
*C. posadasii*	SRP074212	Tucson_15, RMSCC3273	[68, 71]
*C. posadasii*	SRP074212	Tucson_16, RMSCC3275	[68, 71]
*C. posadasii*	SRP074212	Tucson_17, RMSCC3289	[68, 71]
*C. posadasii*	SRP074212	Tucson_18, RMSCC3299	[68, 71]
*C. posadasii*	SRP074212	Tucson_19, RMSCC3300	[68, 71]
*C. posadasii*	SRP074212	Tucson_2, RMSCC3223	[68, 71]
*C. posadasii*	SRP074212	Tucson_20, RMSCC3305	[68, 71]
*C. posadasii*	SRP074212	Tucson_21, RMSCC3317	[68, 71]
*C. posadasii*	SRP074212	Tucson_22, RMSCC3319	[68, 71]
*C. posadasii*	SRP074212	Tucson_23, RMSCC3474	[68, 71]
*C. posadasii*	SRP074212	Tucson_24	[68, 71]
*C. posadasii*	SRP074212	Tucson_3, RMSCC3230	[68, 71]
*C. posadasii*	SRP074212	Tucson_4, RMSCC3231	[68, 71]
*C. posadasii*	SRP074212	Tucson_5, RMSCC3234	[68, 71]
*C. posadasii*	SRP074212	Tucson_6, RMSCC3238	[68, 71]
*C. posadasii*	SRP074212	Tucson_7, RMSCC3240	[68, 71]
*C. posadasii*	SRP074212	Tucson_8, RMSCC3247	[68, 71]
*C. posadasii*	SRP074212	Tucson_9, RMSCC3248	[68, 71]

Whole-genome sequence analyses in Caribbean *Coccidioides* populations suggest that isolates in Venezuela and surrounding areas have been subjected to a recent bottleneck, and thus their populations are less diverse than their conspecific counterparts with more northern distributions [71]. This molecular diversity distribution pattern across geographical space reinforced earlier findings using a microsatellite marker approach by Fisher et al. [39] suggesting that South American populations are younger and less diverse. *C. posadasii* spread to South America 9,000–14,000 years ago, concomitant with human migration patterns and was potentially disseminated by our infected ancestors. While there are fewer genome sequences available and less molecular diversity within *C. immitis* (Table 1), population analyses suggest that there are at least two sub-populations, one in Washington State and the other in Central and Southern California [53, 71]. In their overlapping geographic regions in Southern California and Northern Mexico, we observe signals of introgression between *C. immitis* and *C. posadasii* including within the well-studied isolate *C. immitis* RS. Several considerations should be taken by future researchers when analyzing *Coccidioides* genomic data. First, they should take care to note potential hybridized isolates and the implications for variant identification when selecting reference genomes for alignment-based inferences. Second, while many *Coccidioides* sequences are available on the NCBI Short Read Archive (SRA), a few assemblies including *C. immitis* RS, *C. posadasii* C735, and Silveira are only on the NCBI database (Table 1). Last, there are several *Coccidioides* isolates that have been published under multiple strain names (Table 1). The genome sequencing era has provided ample opportunity to utilize long-standing tools from evolutionary biology to gain insight into *Coccidioides* virulence genetics, providing targets for novel therapeutic design. Cross disciplinary approaches utilizing whole genome sequencing data, evolutionary genomics, and species level diversity are likely to continue unraveling how fungal genomic diversity contributes to our complex interactions with *Coccidioides*.

### Phenotypic Variation

*Coccidioides* genomic content and structure divergence mirrors speciation between *C. immitis* and *C. posadasii* and has resulted in phenotypic differences noted between the species. Environmental conditions in the native geographic range of *C. posadasii* compared to *C. immitis* likely vary in many ways, including soil biochemistry, salinity, temperature, and mammalian host population. Early work on interspecific phenotypic variation showed that single isolates exhibit differential growth in response to salinity, temperature, and humidity [74, 75]. Later, analyses of relatively small populations (<10 individuals) documented differences in fungal growth rates on high salt media, further suggesting that *C. immitis* is more salt tolerant than *C. posadasii* [76]. Indeed, many scientists agree that salinity, temperature, and other environmental variables strongly shape *Coccidioides* physiology and distribution [40, 55•, 77, 78]. The first study to interrogate phenotypic variation in a robust population of 39 *C. posadasii* and 46 *C. immitis* isolates [55•] noted that while *C. posadasii* and *C. immitis* had similar growth rates at 28°C, *C. posadasii* grew significantly faster at 37°C. Earlier work from the same group suggested that *C. immitis* produces spherules synchronously during in vitro culture where *C. posadasii* does not [79]. Though the two species exist in considerably different environments, and phenotypic variation in key virulence traits are currently being investigated, so far there has been no difference clinically between the species [80], and accordingly, currently available diagnostic tools do not differentiate at the species level.

Future efforts focused on publishing robust phenotypic data and the connections to genotypic data will help the *Coccidioides* research community understand connections between life cycle phases spent in the environment and their relationship to virulence factors observed in clinics. Further, efforts focused on molecular diversity underpinning sporulation and dispersal dynamics, range expansion, and host affiliation will enable robust predictive modeling and inform pandemic preparedness.

### Virulence Genetics

The *Coccidioides* genome contains ~7,000–9,000 protein coding genes, and nearly half lack functional gene annotation predictions [71]. While it is clear that expansions and contractions of gene families have enabled *Coccidioides* and other fungi in Onygenales to target animal cell wall degradation rather than plant cell walls, functionality of the *Coccidioides* genome as a whole remains relatively poorly understood. It is an exciting time to be a *Coccidioides* genomics researcher, as the ~50% of genes with annotations based on homology to known orthologs in other fungi may lack relevance due to the unique cellular structures *Coccidioides* produced during infections, including the spherule. As in all genome annotation endeavors, targeted gene deletion is needed to confirm functions based on computational gene annotation and functional and genomic screens. In total, less than 10 genes have been functionally characterized in *Coccidioides*, many of which appear to be related to virulence [81–87]. For example, deletion of urease gene (URE) partially reduced ammonia production and increased mice survival by 60% [88]. Further, double deletion of urease gene (URE) Ureidoglycolate hydrolase (Ugh) resulted in even lower extracellular ammonia levels and increased mice survival to 90%. These findings are supported by direct measurements, transcriptome, and volatome analyses which show that ammonia production contributes to virulence during spherule development and rupture. In another multiple gene deletion strategy, Dr. Chiung-Yu Hung et al. created a completely avirulent strain of *Coccidioides* as a vaccine candidate through partial deletion of chitinase genes among others (*cts2/ard1/cts3* or ΔT) [83]. Loss of virulence appears to be associated with genes responsible for intracellular chitin remodeling leading to multiple changes at a transcriptional level preventing spherule rupture.

To date, all gene deletions have been completed within the *C. posadasii*, wildtype C735 background and one in *C. posadasii* strain, Silveira; therefore, intra- or interspecies virulence gene sequence variation has yet to be assessed (Table 1) [81]. However, the broad spectrum of disease symptoms in humans coupled with strong regional population genetic variation among *Coccidioides* isolates suggests that virulence mechanisms and severity may co-vary by species or region. Investigations by Dr. Emily Whiston showed that transcriptional patterns are generally shared between species but approximately 500 genes are differentially expressed during parasitic phase spherule growth, suggesting unique gene usage between species [73]. Phenotypic differences have been documented between species, including differential thermotolerance behavior [89], saline tolerance [76], and spherule growth patterns [79]. However, the underlying gene functions associated with these phenotypes remain largely unknown. Lewis et al. identified that mice infected with equivalent fungal inoculum had increased fungal burden and *il-1*β response to *C. posadasii* Silveira isolate but not to *C. immitis* 2006 or RS [90], potentially indicating hypervirulence in some isolates. Linking virulence to gene sequence and function is a top research priority. Progress in this realm is underscored by variation in the gene sequence of SOWgp (spherule outer wall protein), an extracellular glycoprotein which binds to host cells. The SOWgp deletion mutant demonstrated decreased binding in vitro and virulence in vivo. While the current SOWgp mutant was created in the *C. posadasii* background, molecular techniques revealed that other strains of *C. posadasii* showed variation in SOWgp protein size and quantity of repeats, which could potentially alter function [86], and SOWgp sequence variation within *C. immitis* is unknown. As previously mentioned, a successful gene editing strategy for *C. immitis* has not been developed, thus hindering gene function comparisons between species.

In an era of advanced genetic and genomic technologies, it may come as a surprise that such a small handful of genes have been functionally characterized and primarily in one *C. posadasii* isolate. Research progress in this area has been severely limited by development of genome editing tools for *Coccidioides* [91]. Current strategies use either introduction of linear DNA to susceptible protoplasts or *Agrobacterium*-mediated transformation, the latter of which appears more efficient. Challenges for efficient transformation include poly-nucleate arthroconidia, persistence of heterokaryons following transformation selection, and logistics of biosafety level three (BSL3) laboratory experiments [85, 92]. Creation of autotrophic mutants [93] or CRISPR Cas9 [94] technologies have proven successful approaches in other fungal pathogens but have yet to be applied to *Coccidioides*.

### Host Immune Response and Development of a Vaccine

The progress of vaccine and immunology research in the *Coccidioides* field is largely thanks to the women researchers in the field. There are some excellent women-led review articles highlighting the recent advances in the immune response, host-pathogen interactions, and development of a *Coccidioides* vaccine [95•, 96–98, 99•]. In this section, we will discuss briefly what is known about the host immune response to *Coccidioides* and the development of a vaccine to combat coccidioidomycosis.

### Host Immune Response

The innate immune response is the first line of defense against many fungal pathogens, especially macrophages and neutrophils. Dr. Chiung-Yu Hung has shown that neutrophils are increased significantly during a *Coccidioides* infection [100]. Her work has shown that the role of neutrophils is not a one size fits all scenario. On one hand, mice depleted of neutrophils during a primary infection are just as susceptible as wild-type mice [101]. Mice vaccinated with a live, attenuated (ΔT vaccine) strain require neutrophils to be protected against challenge with *Coccidioides*. Vaccination studies have also shown an increased presence of macrophages in vaccinated mice compared to unvaccinated mice after challenge with *Coccidioides* [100]. Furthermore, studies show that mouse peritoneal macrophages produce tumor necrosis factor alpha, TNF-α, when stimulated with *Coccidioides* spherules [102]. Pattern recognition receptors (PRRs) such as toll-like receptors (TLRs) and C-type Lectin receptors interact with cell components or pathogen-associated molecular patterns (PAMPs) to detect *Coccidioides* [103]. Using peritoneal macrophages from wild-type and knockout mice (TLR2^-/-^ and MyD88^-/-^), studies have shown that host response to *Coccidioides* spherules relies on TLR2, myeloid differentiation factor 88 (MyD88), and Dectin-1 [104]. Furthermore, studies suggest that alternative splicing of Dectin-1 in C57BL/6 mice causes increased susceptibility to coccidioidomycosis [105]. Studies performed by Dr. Althea Campuzano and colleagues has shown that macrophages isolated from Dectin-1^-/-^, Dectin 2^-/-^, and CARD9^-/-^ mice produced less inflammatory cytokines in response to the GCP-rCpa1 vaccine compared to wild-type mice [106•]. Additionally, these studies demonstrate less efficacy of the GCP-rCpa1 vaccine in Dectin-1^-/-^, Dectin 2^-/-^, and CARD9^-/-^ mice compared to vaccinated wild-type mice. Although much remains to be done, these studies demonstrate the crucial role of the innate immune response to protect against coccidioidomycosis.

T cells have been shown to be critical for protection against coccidioidomycosis. CD4^+^T cell deficiency leads to increased susceptibility to a *Coccidioides* infection [107]. Furthermore, CD4^+^T cells differentiate into distinct lineages based on cytokines produced in response to a pathogen. A T cell helper 1 (Th1) response is associated with cytokines such as IL-12 and IFN-γ and has been shown to be important for protection against coccidioidomycosis [83, 108], while a Th2-type response, activated by cytokines such as IL-4 and IL-5, has been shown to downregulate the immune response during a *Coccidioides* infection [83]. Th17 responses, activated by IL-17 and IL-22, have been shown to be critical for protection against coccidioidomycosis [109]. Studies to understand the host immune response to *Coccidioides* mainly use *C. posadasii* as a model and assume *C. immitis* to have a similar response; however, more studies are needed to discern this.

### Vaccine Development

**Figure 2.**
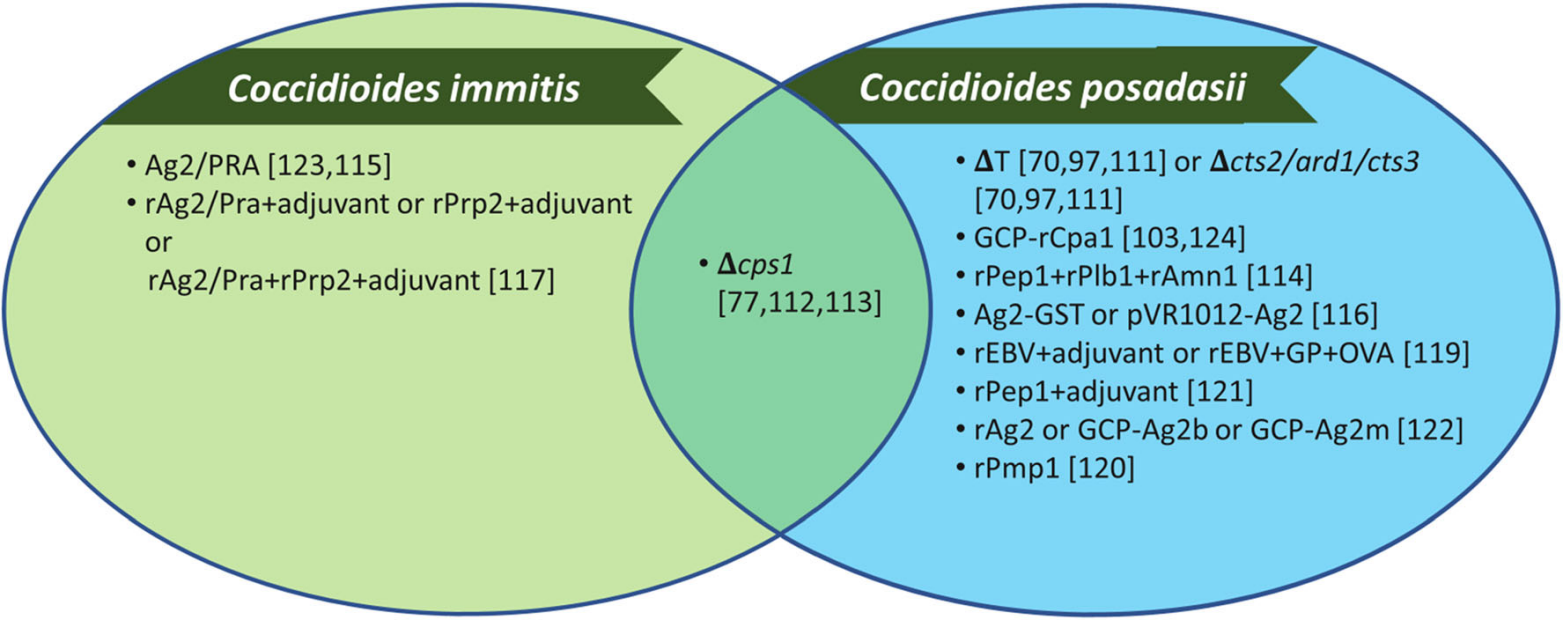
Species tested with coccidioidomycosis vaccine candidates. Vaccination studies against coccidioidomycosis mainly test against *C. posadasii* infections.

There is no clinically available vaccine against any human fungal pathogen. Interestingly, a *Candida albicans* vaccine, NDV-3A, demonstrates promise in clinical trials against recurrent vulvovaginal candidiasis [110], the potential to prevent colonization on medical devices [111], and even prevent against *C. auris* infection [112]. Many live attenuated strains have demonstrated protection against coccidioidomycosis; however, live vaccines are not preferred due to their potential safety concerns [113]. Some of the live attenuated candidates include ΔT (also known as Δ*cts2/ard1/cts3*) [83, 100, 114] and ΔCPS1 [81, 115]. Dr. Lisa Shubitz and colleagues are developing ΔCPS1 as a vaccine candidate to prevent infection due to *C. posadasii* in dogs [116]. Many studies have identified protective antigens (Pep1, Plb, Amn1, Ag2/Pra, Cs-Ag, Pmp1, Prp2, Ure, and Gel1) that could be used in a recombinant protein vaccine [117–125]. Although these studies demonstrated varying levels of protection, the use of multivalent vaccines shows more efficacy than a single peptide vaccine against coccidioidomycosis [117, 126]. Dr. Chiung-Yu Hung and colleagues have developed a multivalent vaccine encapsulated in glucan-chitin particles, GCP-rCpa1, that has demonstrated increased survival, significantly reduced fungal burden, and showed a protective Th1 and Th17 response against *C. posadasii* in a murine model of coccidioidomycosis [127]. Furthermore, recent studies have shown that protection mediated by the GCP-rCpa1 vaccine is due to enhancement of Th17 responses and activation of CARD9-associated Dectin-1 and Dectin-2 signal pathways [106•]. A majority of the vaccination studies against coccidioidomycosis demonstrate protection mainly against *C. posadasii* infections [81, 83, 100, 106•, 114, 116, 117, 119, 122–127]. There are a couple studies that demonstrate protection against *C. immitis* [118, 120] and only one study to demonstrate protection against both species [115] (Figure 2).

### Antifungal Drugs

Antifungal medications from the triazole and polyene classes are the mainstay of treatment for coccidioidomycosis. Dr. Marley Van Dyke et al. recently summarized the agents with coverage of *Coccidioides* along with potential novel agents [95•]. While robust, prospective studies investigating the most appropriate treatment are lacking, fluconazole, an oral and intravenous triazole, is the most prescribed treatment and recommended as the first-line agent for primary pulmonary infection [70]. It is important to note that treatment is not always warranted, especially in the setting of uncomplicated, mild disease [70]. In cases of severe infection with dissemination and/or meningitis, amphotericin B, an intravenous polyene agent, is used as induction therapy. Amphotericin B requires intense laboratory monitoring and is typically prescribed with the aid of an infectious diseases specialist, as it can cause renal and hepatotoxicity along with infusion reactions [95•]. Investigation of novel antifungal agents for treatment of coccidioidomycosis will be crucial to expand our arsenal for disease treatment.

## Conclusion

Women have pioneered *Coccidioides* and coccidioidomycosis research in the past, shedding light on previous large questions in the field. While numerous big questions remain regarding the environmental, genetic, and clinical aspects of this disease, it is important that the *Coccidioides* community continues to acknowledge and support the work of women, as well as push for further diversity and inclusion regarding this disease. Doing so will strengthen the scientific research being done on *Coccidioides* and likely mitigate some of the negative health outcomes from coccidioidomycosis.

## Data Availability

Not applicable.
